# In This Issue

**DOI:** 10.1002/cfg.112

**Published:** 2001-10


**Comparative and Functional Genomics**
*Comp Funct Genom* 2001; **2**: 273–274.
**DOI:** 10.1002/cfg.112

### In this issue

### Identification of membrane proteins in the hyperthermophilic archaeon *Pyrococcus furiosus* using proteomics and prediction programs

James Holden *et al.*, have used a proteomic approach to identify 32 *Pyrococcus furiosus* membrane proteins. Using these proteins they then tested the performance of six sub-cellular location prediction programs, achieving a consensus result for 'membrane protein' for 23 of the 32 proteins.

### Sphingoid base metabolism in yeast: mapping gene expression patterns into qualitative metabolite time course predictions

Tomas Radivoyevitch attempts to answer the question: Can qualitative metabolite time course predictions be inferred from measured mRNA expression patterns? Using a simple kinetic model of sphingoid base metabolism in yeast, and modulating enzyme activities in line with changes observed in mRNA levels in heat-shocked yeast he has predicted alterations in sphingoid base and ceramide levels which match experimental data.

### Special Section: The ESF programme on Integrated Approaches for Functional Genomics workshop 'Proteomics: Focus on Protein Interactions'

### Conference Editorial

Gianni Cesareni introduces this special section on the first workshop of the ESF programme on Integrated Approaches to Functional Genomics.

### Protein arrays: issues to be addressed

Michael Taussig, Chairman of the ESF programme on Integrated Approaches for Functional Genomics brings us his introductory comments, made as Chairperson for the session on 'Peptide and protein chips'.

### How useful will functional proteomics data be?

Pierre Legrain discusses the importance of comparing protein interaction networks, highlighting issues relating to standardization that require serious consideration by the community. He also comments on common problems with the datasets produced by global approaches to protein-protein interactions.

### Mapping protein-protein interactions with combinatorial peptides

Brian Kay reviews the use of phage-displayed combinatorial peptides in the study of protein-protein interactions, giving several examples of successful studies.

### Synthetic peptide and protein domain arrays prepared by the SPOT technology

Jens Schneider-Mergener presents the SPOT technology for highly parallel synthesis of peptides and protein domains on flat surfaces, which is being used to produce protein arrays.

### Extracting information automatically from biological literature

Christian Blaschke *et al.* review the progress made so far in the application of information extraction (IE) technology to molecular biology, profiling two existing automated approaches.

### iSPOT: a web tool for the analysis and recognition of protein domain specificity

Barbara Brannetti *et al.* present iSPOT, a web tool for predicting interactions between protein domains. Originally developed for application to the SH3 domain family, the procedure has now been modified for use on PDZ domains and MHC class I molecules.

### Conference Report

Concluding this special section, Joan Marsh and Michael Taussig bring us an in-depth report on the ESF workshop, focusing on those presentations not represented in the special section.

Copyright © 2001 John Wiley & Sons, Ltd.

### Meeting Review: Bioinformatics Open Source Conference (BOSC2001)

Cara Woodwark reports on this satellite meeting of ISMB2001, which was organized around existing open source bioinformatics projects, encouraging the exchange of ideas between projects.

### Meeting Review: Intelligent Systems in Molecular Biology (ISMB2001)

Cara Woodwark brings us a report from the 9$^{th}$ ISMB international conference, with sessions on 'Protein Structure and Folding', 'Sequence Motifs, Alignments and Families', 'Networks and Modeling', 'Gene Structure, Regulation and Modeling', 'Methods', and 'Sequence and Phylogeny'.

### Protein-protein interactions on the web

We present a brief guide to resources on the Internet relating to protein-protein interactions. These include databases containing experimentally verified and computationally inferred physical and functional interactions, tools for predicting interactions and extracting information on interactions from the literature, and organism specific databases.

*The* **Genomics** *Website at Wiley*

**www.wiley.co.uk/genomics**

The **Genomics** website at Wiley is a **DYNAMIC** resource for the genomics community, offering **FREE** special feature articles and new information **EACH MONTH**.

Find out more about our new journals *Comparative and Functional Genomics,* and *Proteomics.*

Visit the **Library** for hot books in Genomics, Bioinformatics, Molecular Genetics and more.

Click on **Journals** for information on all our up-to-the minute journals, including: *Genesis, Bioessays, Journal of Mass Spectrometry, Gene Function and Disease, Human Mutation, Genes, Chromosomes and Cancer* and the *Journal of Gene Medicine.*

Let the **Genomics** website at Wiley be your **guide** to genomics-related web sites, manufacturers and suppliers, and a calendar of conferences.

WILEY

2380

Copyright © 2001 John Wiley & Sons, Ltd.

